# Supplementary material for: Local membrane source gathering by p62 body drives autophagosome formation
Source: Nat Commun. 2023 Nov 13;14:7338. doi: 10.1038/s41467-023-42829-8 (PMC10643672; doi:10.1038/s41467-023-42829-8)
Supplement: Supplementary file 1 — Supplementary Information [file 41467_2023_42829_MOESM1_ESM.pdf]

SUPPLEMENTARY INFORMATION

Local membrane source gathering by p62 body drives autophagosome formation

Xuezhao Feng[1,2,†], Daxiao Sun[3,†,*], Yanchang Li[4,†], Jinpei Zhang[1,2,†], Shiyu Liu[1,2], Dachuan Zhang[5], Jingxing Zheng[5], Qing Xi[1,2], Haisha Liang[5], Wenkang Zhao[5], Ying Li[5], Mengbo Xu[1,2], Jiayu He[1,2], Tong Liu[1,2], Ayshamgul Hasim[2,6], Meisheng Ma[7], Ping Xu[4,*] and Na Mi[1,2,*]

Supplementary Information associated with this article include:

Supplementary Figs. 1-14
Supplementary Tables 1-5 (attached datasets)

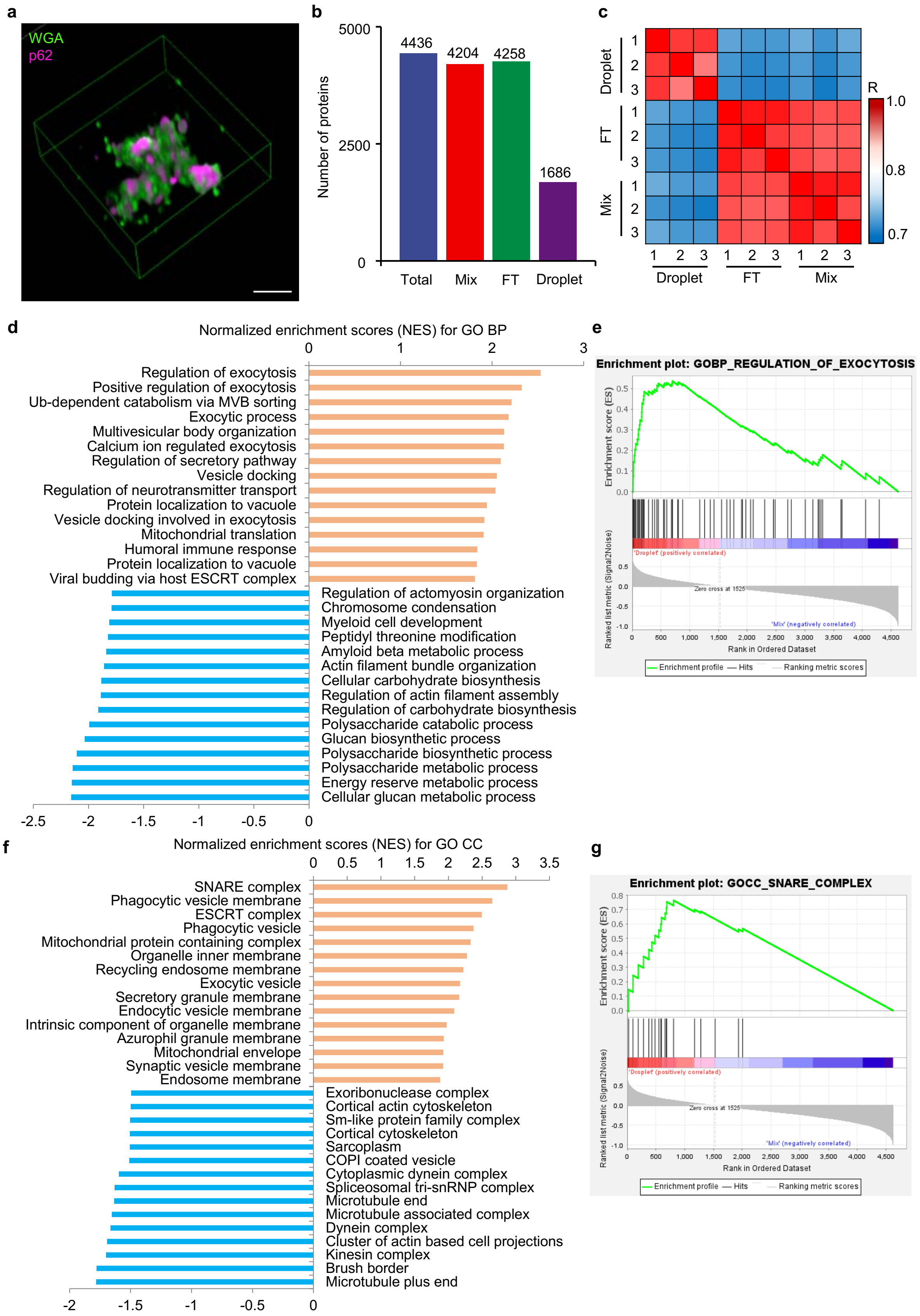
a
WGA
p62
b
5000
2500
0
Number of proteins
4436
4204
4258
1686
Total
Mix
FT
Droplet
c
Droplet
FT
Mix
1
2
3
R
1.0
0.8
0.7
d
Normalized enrichment scores (NES) for GO BP
Regulation of exocytosis
Positive regulation of exocytosis
Ub-dependent catabolism via MVB sorting
Exocytic process
Multivesicular body organization
Calcium ion regulated exocytosis
Regulation of secretory pathway
Vesicle docking
Regulation of neurotransmitter transport
Protein localization to vacuole
Vesicle docking involved in exocytosis
Mitochondrial translation
Humoral immune response
Protein localization to vacuole
Viral budding via host ESCRT complex
Regulation of actomyosin organization
Chromosome condensation
Myeloid cell development
Peptidyl threonine modification
Amyloid beta metabolic process
Actin filament bundle organization
Cellular carbohydrate biosynthesis
Regulation of actin filament assembly
Regulation of carbohydrate biosynthesis
Polysaccharide catabolic process
Glucan biosynthetic process
Polysaccharide biosynthetic process
Polysaccharide metabolic process
Energy reserve metabolic process
Cellular glucan metabolic process
-2.5 -2 -1.5 -1 -0.5 0
0 1 2 3
e
Enrichment plot: GOBP_REGULATION_OF_EXOCYTOSIS
Enrichment score (ES)
'Droplet' (positively correlated)
Zero cross at 1525
'Mix' (negatively correlated)
Ranked list metric (Signal2Noise)
Rank in Ordered Dataset
Enrichment profile — Hits — Ranking metric scores
f
Normalized enrichment scores (NES) for GO CC
0 0.5 1 1.5 2 2.5 3 3.5
SNARE complex
Phagocytic vesicle membrane
ESCRT complex
Phagocytic vesicle
Mitochondrial protein containing complex
Organelle inner membrane
Recycling endosome membrane
Exocytic vesicle
Secretory granule membrane
Endocytic vesicle membrane
Intrinsic component of organelle membrane
Azurophil granule membrane
Mitochondrial envelope
Synaptic vesicle membrane
Endosome membrane
Exoribonuclease complex
Cortical actin cytoskeleton
Sm-like protein family complex
Cortical cytoskeleton
Sarcoplasm
COPI coated vesicle
Cytoplasmic dynein complex
Spliceosomal tri-snRNP complex
Microtubule end
Microtubule associated complex
Dynein complex
Cluster of actin based cell projections
Kinesin complex
Brush border
Microtubule plus end
-2 -1.5 -1 -0.5 0
g
Enrichment plot: GOCC_SNARE_COMPLEX
Enrichment score (ES)
'Droplet' (positively correlated)
Zero cross at 1525
'Mix' (negatively correlated)
Ranked list metric (Signal2Noise)
Rank in Ordered Dataset
Enrichment profile — Hits — Ranking metric scores

Supplementary Fig. 1 Membrane vesicle associated proteins are highly enriched in p62 droplet by proteomic analysis.

a, p62 droplet formation by 3D fluorescence imaging. mCherry-p62 droplet and cytosol were labeled with wheat-germ agglutinin (WGA) -Alexa 488.

b, Numbers of identified proteins in different fractions with MS2 spectrum evidence.

c, Pearson correlation coefficients of three biological replicates between Mix, FT and droplet.

d, Gene Set Enrichment Analysis (GSEA) for GO biological processes (BP) of proteins enriched in droplet (up, orange panel) and Mix (down, blue panel).

e, Enrichment plots for the top two data set enriched in GSEA BP analysis. Gene set of "Regulation of exocytosis" was significantly enriched in droplet.

f, GSEA for GO cellular components (CC) of proteins enriched in droplet (up, orange panel) and Mix (down, blue panel).

g, Enrichment plot for the top data set enriched in GSEA CC analysis. Gene set of "SNARE complex" was significantly enriched in droplet.

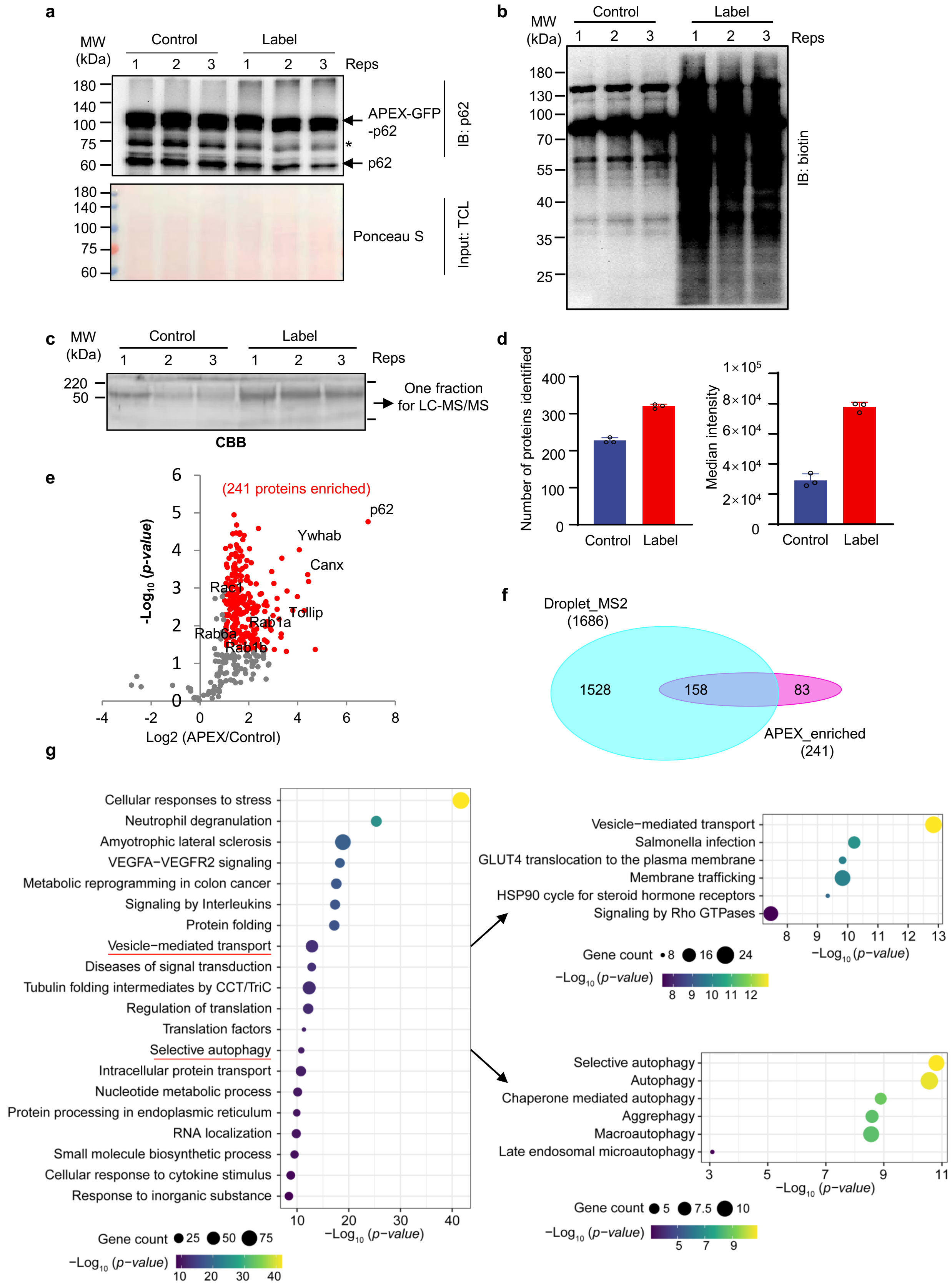

a
MW (kDa)
Control
Label
1 2 3 1 2 3 Reps
180
140
100
75
60
APEX-GFP -p62
*
p62
IB: p62
Ponceau S
Input: TCL
b
Control
Label
Reps
180
130
100
70
55
40
35
25
IB: biotin
c
One fraction for LC-MS/MS
220
50
CBB
d
Number of proteins identified
Median intensity
Control
Label
e
(241 proteins enriched)
p62
Ywhab
Canx
Rac1
Tollip
Rab1a
Rab6a
Rab1b
-Log10 (p-value)
Log2 (APEX/Control)
f
Droplet_MS2 (1686)
1528
158
83
APEX_enriched (241)
g
Cellular responses to stress
Neutrophil degranulation
Amyotrophic lateral sclerosis
VEGFA−VEGFR2 signaling
Metabolic reprogramming in colon cancer
Signaling by Interleukins
Protein folding
Vesicle−mediated transport
Diseases of signal transduction
Tubulin folding intermediates by CCT/TriC
Regulation of translation
Translation factors
Selective autophagy
Intracellular protein transport
Nucleotide metabolic process
Protein processing in endoplasmic reticulum
RNA localization
Small molecule biosynthetic process
Cellular response to cytokine stimulus
Response to inorganic substance
Gene count
−Log10 (p-value)
Vesicle−mediated transport
Salmonella infection
GLUT4 translocation to the plasma membrane
Membrane trafficking
HSP90 cycle for steroid hormone receptors
Signaling by Rho GTPases
Selective autophagy
Autophagy
Chaperone mediated autophagy
Aggrephagy
Macroautophagy
Late endosomal microautophagy

Supplementary Fig. 2 APEX2-based proximity profiling of p62 adjacent proteins.

a, Western blot analysis of the endogenous p62 and transfected APEX2-GFP-p62. Three biological replicates were performed for control and label groups, respectively. The control group was treated in parallel but without H_2O_2 stimulus compared to label group. The asterisk might indicate unspecific signal.

b, Western blot analysis after APEX2-p62 activation and streptavidin pull-down of biotinylated proteins. Three biological replicates were performed.

c, SDS-PAGE separation of the control and label samples and digested by trypsin. Each sample was cut into one fraction and analyzed by LC-MS/MS.

d, Number of proteins identified and median intensity of the control (blue bar) and label (orange bar) experiment.

e, Dot plot showing the APEX-enriched p62 adjacent proteins (highlighted in red dots) over the control.

f, Venn diagram showing the overlap between APEX-enriched p62 adjacent proteins and p62 droplet proteins.

g, Metascape enrichment of the proteins enriched in both the droplet and the APEX labeling group (n=158). The top 20 enriched main cluster terms of biological pathways were presented. The sub clusters of "*Salmonella* infection" (purple) and "Selective autophagy" (orange) were shown.

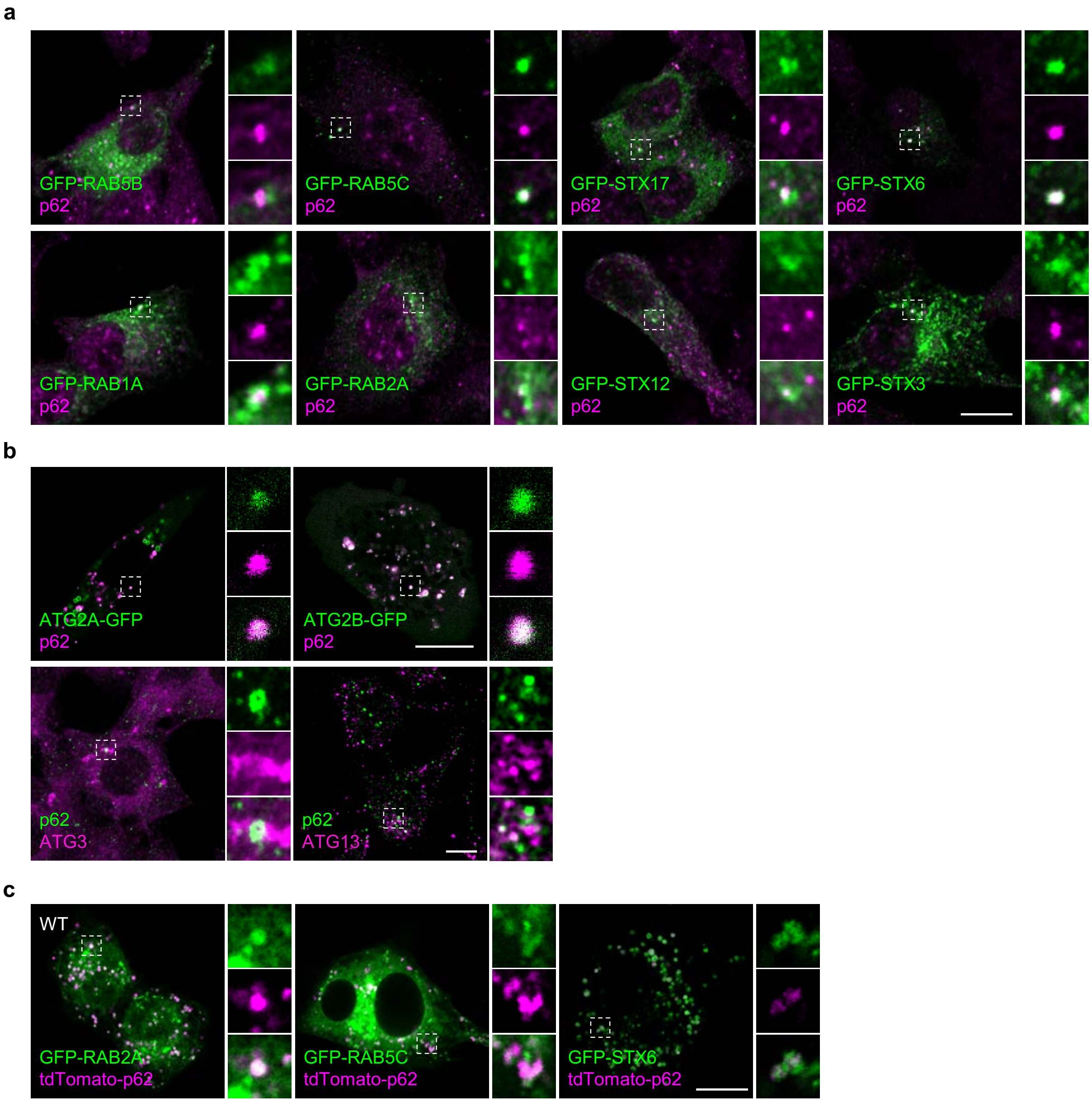

Supplementary Fig. 3 Validation of colocalization of membrane vesicle proteins and ATG proteins with p62 bodies.

a, EGFP-tagged RAB5B, RAB5C, RAB1A, RAB2A, STX17, STX6, STX12 and STX3 were transiently expressed in NRK cells, starved for 4h, and cells were fixed and stained with antibodies against GFP and p62. Scale bar, 10 µm.

b, EGFP-tagged ATG2A, ATG2B were transiently expressed in NRK cells. The cells were starved for 4 h, then stained with antibodies against GFP and p62 (upper panels). NRK cells starved for 4 h were stained with antibodies against p62 and ATG3 or ATG13. Scale bar, 10 µm.

c, EGFP-tagged RAB5C, RAB2A and STX6 were transiently co-expressed with tdTomato-p62 in NRK cells. Scale bar, 10 µm.

a

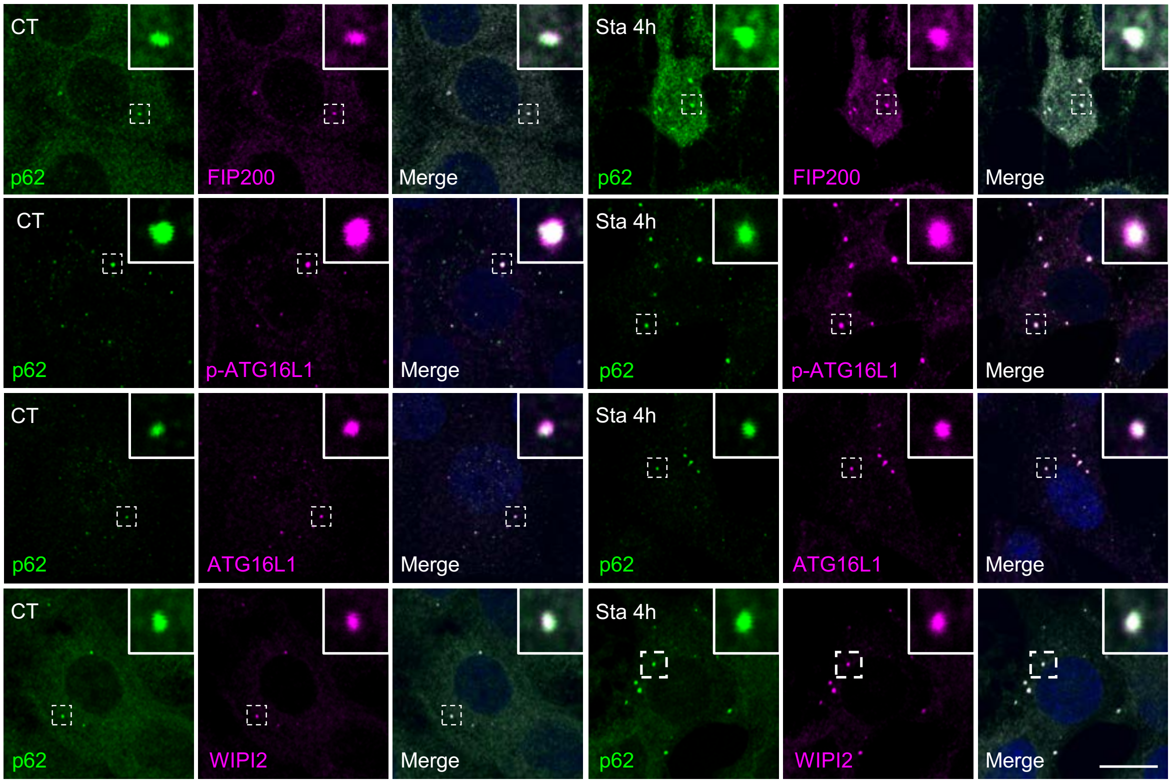

b

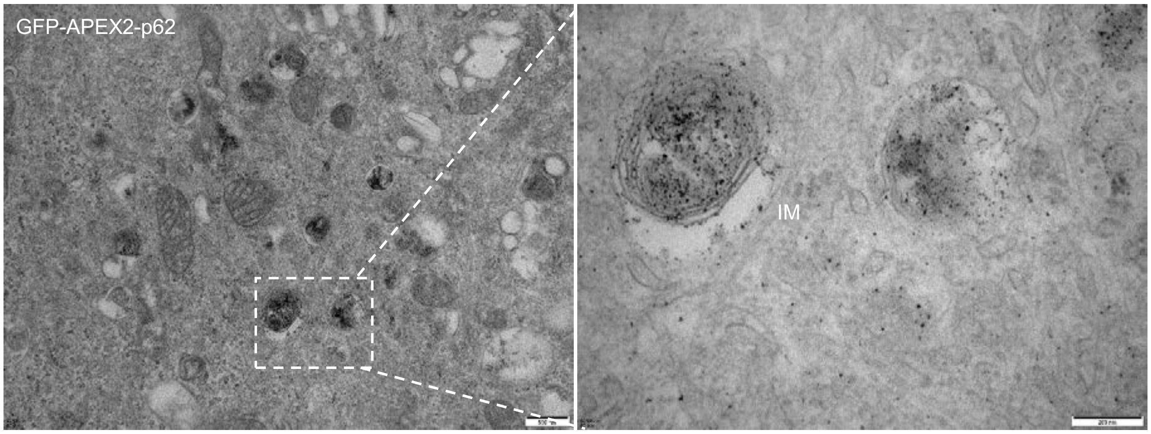

Supplementary Fig. 4 The colocalization status of autophagic proteins with p62 bodies and isolation membrane formed around p62 bodies.

a, NRK cells were starved for 4 h, and then stained with antibodies against FIP200, p-ATG16L1, ATG16L1, WIPI2 and p62. Scale bar, 10 µm. CT, control; Sta, starvation.

b, TEM image showing the DAB staining pattern in WT cells transiently transfected with GFP-APEX2-p62. Scale bar, 500nm, 200nm. IM, isolation membrane.

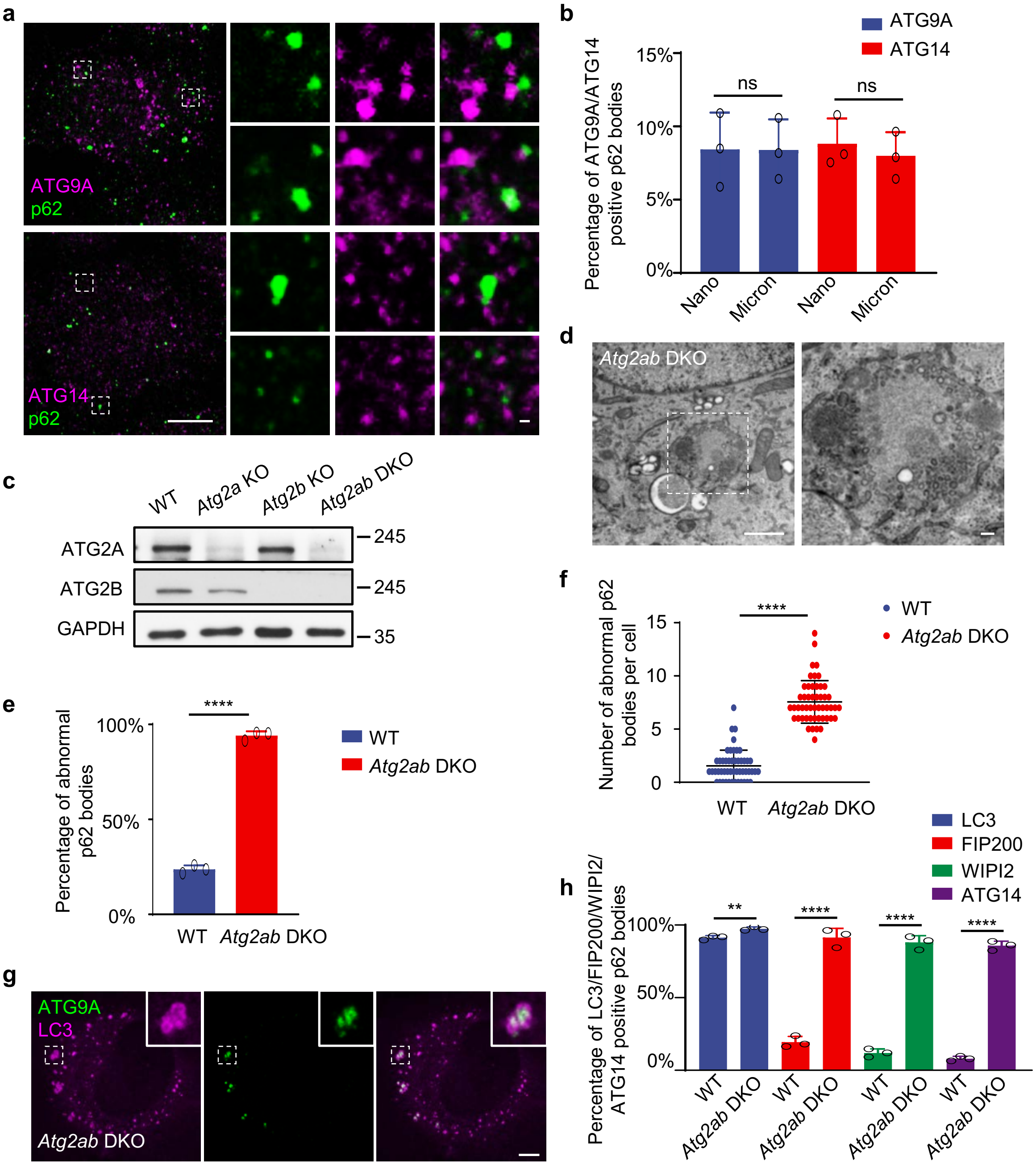

Supplementary Fig. 5 Colocalization of the core autophagy machinery with p62 bodies in *Atg2ab* DKO cells.

a, NRK cells were starved for 4 h, and then stained with antibodies against ATG9A, ATG14 and p62. The left panel shows the co-localization of ATG9A, ATG14 and p62. Right panels show enlarged p62 structures that were either ATG9A, ATG14 and p62 positive. Scale bar, 10 µm.

b, The percentage of ATG9A-, ATG14- and p62-positive (micron-scale, Micron) or -negative (nanoscale, Nano) p62 bodies was quantified in cells from **a**. Data are presented as mean ± SD, n=3 independent experiments; 100 puncta were assessed per independent experiment. *P* values were calculated using the two-tailed, unpaired t-test. ns means not significant.

c, Immunoblot analysis of wild-type (WT) and *Atg2ab* DKO, *Atg2a* KO or *Atg2b* KO cells with the Atg2a and Atg2b antibodies.
d, The vesicles around of p62 bodies were observed by TEM in the *Atg2ab* DKO cells. The right panel shows an enlarged p62 body and vesicles. Scale bar, 1 μm.
e, The percentage of abnormal p62 bodies in either WT and *Atg2ab* DKO cells. Data are presented as mean ± SD, n=3 independent experiments; ~100 cells were assessed from 3 independent experiments. *P* values were calculated using the two-tailed, unpaired t-test, $^{**}P < 0.01$ $^{****}P < 0.0001$.
f, The numbers of abnormal p62 bodies in either WT and Atg*2ab* DKO per cell were quantified. (n=50 individual cells). *P* values were calculated using the two-tailed, unpaired t-test, $^{**}P < 0.01$ $^{****}P < 0.0001$.
g, Cells were transfected with GFP-ATG9A together with mCherry-LC3, and then imaged. Regions containing LC3 foci that co-localize with ATG9A are outlined with white dashed lines and are magnified in the insets. Scale bars, 5μm.
h, The percentage of indicated protein-positive p62 bodies in either WT and *Atg2ab* DKO cells. Data are presented as mean ± SD, n=3 independent experiments; 100 puncta were assessed per independent experiment. *P* values were calculated using the two-tailed, unpaired t-test, $^{**}P < 0.01$ $^{****}P < 0.0001$.

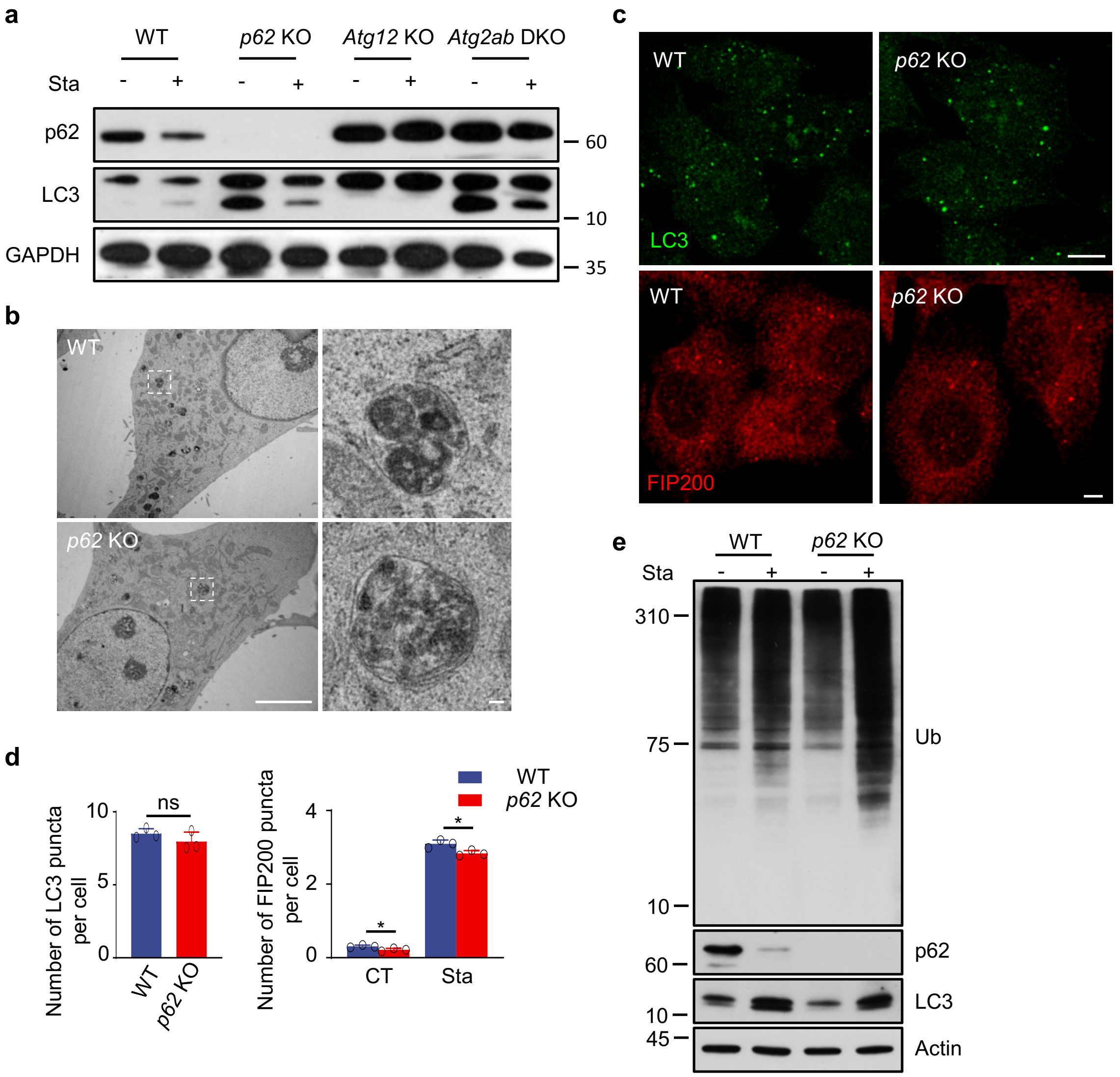

Supplementary Fig. 6 *p62* knockout mainly affects selective rather than non-selective autophagy.

a, NRK, *p62* KO, *Atg12* KO and *Atg2ab* DKO cells were starved with DPBS (Sta) for 4 h, and then the cell lysates were analysed by Western blot with antibodies against p62, LC3 and GAPDH.

b, The autophagosomes or autolysosomes were observed by TEM in NRK WT and *p62* KO cells. Scale bar, 2 μm.

c, WT and *p62* KO cells were starved with DMEM medium for 4 h, and then stained with antibodies against LC3 or FIP200. Scale bars, 10μm.

d, The number of LC3 or FIP200 puncta was quantified in images from **c**. Data are presented as mean ± SD, n=3 independent experiments; 50 cells were assessed per independent experiment. The *P* value was calculated using the two-tailed, unpaired t-test. *$P < 0.05$.

e, WT and *p62* KO cells were starved for 4 h, and the cell lysates were analyzed by Western blot with antibodies against ubiquitin, Actin and LC3.

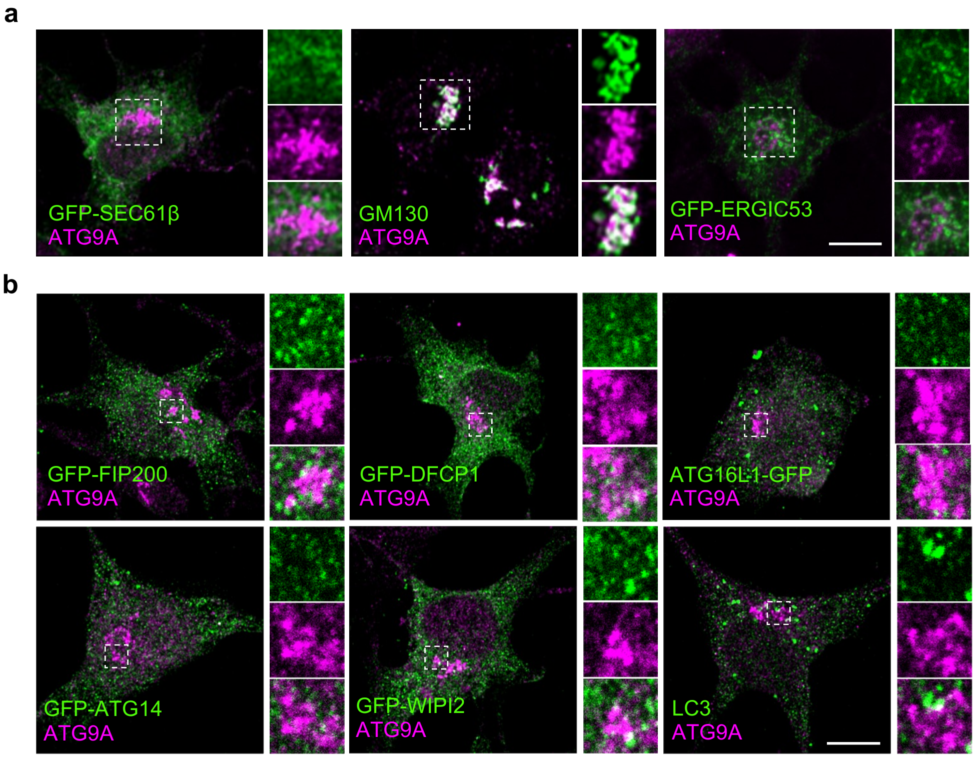

Supplementary Fig. 7 Aberrant localization of ATG9 vesicles and other ATG proteins in p62 KO cells.

a, EGFP-tagged SEC61β or GFP-ERGIC53 was transiently expressed in *p62* KO cells. The cells were stained with antibodies against GFP and ATG9A. *p62* KO cells were stained with antibodies against GM130 and ATG9A. Scale bar, 10 µm.

b, EGFP-tagged FIP200, DFCP1, ATG16, ATG14 or WIPI2 were transiently expressed in *p62* KO cells, and the cells were stained with antibodies against GFP and ATG9A. *p62* KO cells were starved and stained with antibodies against LC3 and ATG9A. Scale bar, 10 µm.

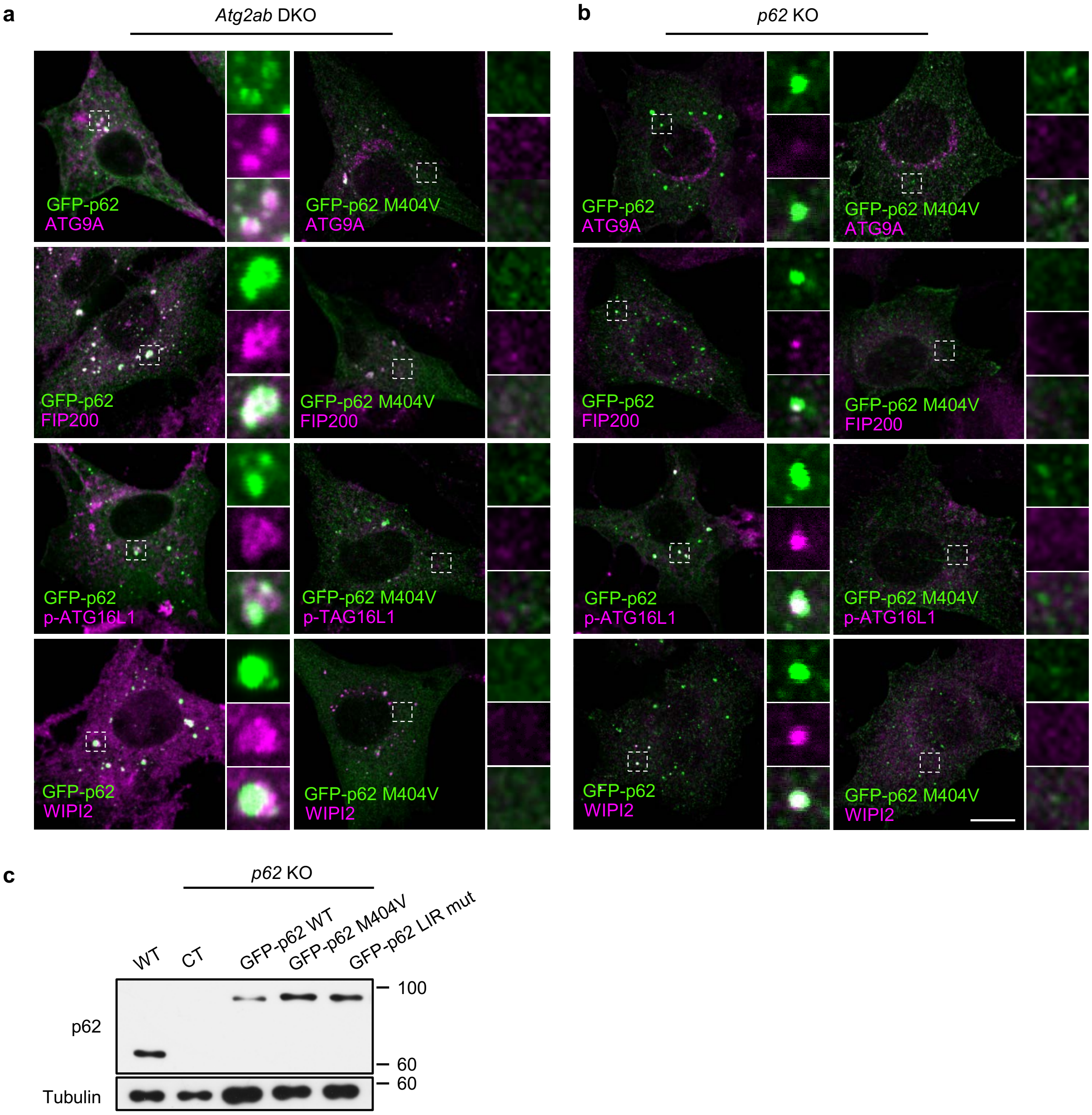

Supplementary Fig. 8 The recruitment of membrane vesicles and autophagy proteins by phase separation deficient p62 M404V mutant.

a-b, EGFP-tagged p62 or p62 M404V was transiently expressed in *Atg2ab* DKO cells (a) and *p62* KO cells (**b**). The cells were then stained with antibodies against GFP, ATG9A, FIP200, p-ATG16L1 and WIPI2. Scale bar, 20 µm.

c, Western blot analysis of transient expression level of EGFP-tagged p62 or p62 M404V or p62 LIR mut in *p62* KO cells with antibodies against p62 and Tubulin.

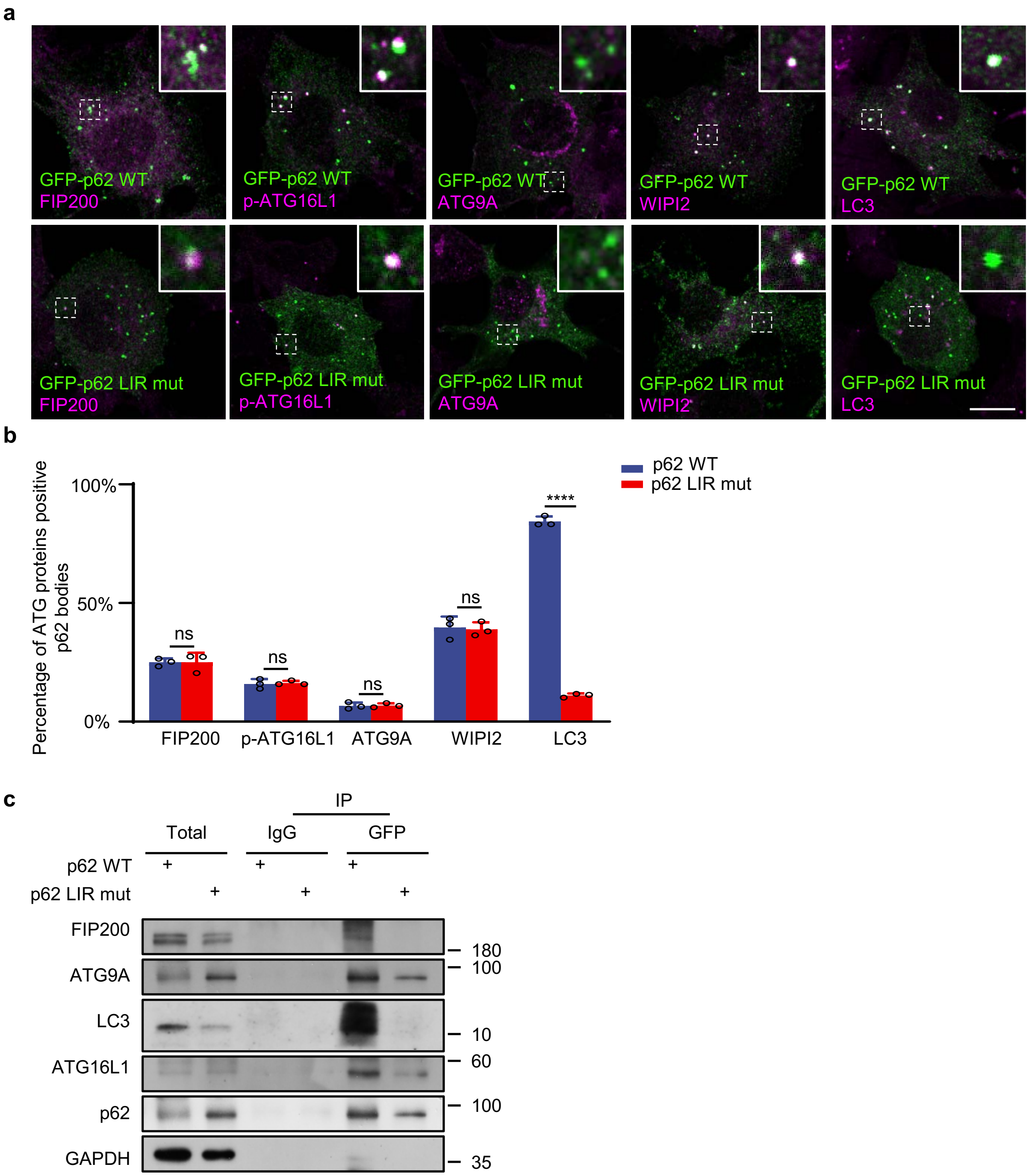

Supplementary Fig. 9 The recruitment of membrane vesicles and autophagy proteins by p62 LIR mutant.

a, EGFP-tagged p62 or p62 LIR mutant were transiently expressed in NRK cells, and cells were stained with antibodies against GFP and FIP200, p-ATG16L1, ATG9A, WIPI2 or LC3. Scale bar, 10 μm.

b, The percentage of indicated protein-positive p62 bodies were quantified from data in **a**. Data are presented as mean ± SD, n=3 independent experiments; 100 puncta were assessed per

independent experiment. The *P* value was calculated using the two-tailed, unpaired t-test. ****$P < 0.0001$, ns means not significant.

c, EGFP-tagged p62 or EGFP-tagged p62 LIR mut was transiently expressed in *p62* KO cells. Immunoprecipitation was performed with IgG or GFP antibodies and protein A/G magnet beads, and the interacting proteins were analyzed by immunoblotting with FIP200, ATG9A, GFP, ATG16L1, LC3 and GAPDH antibodies.

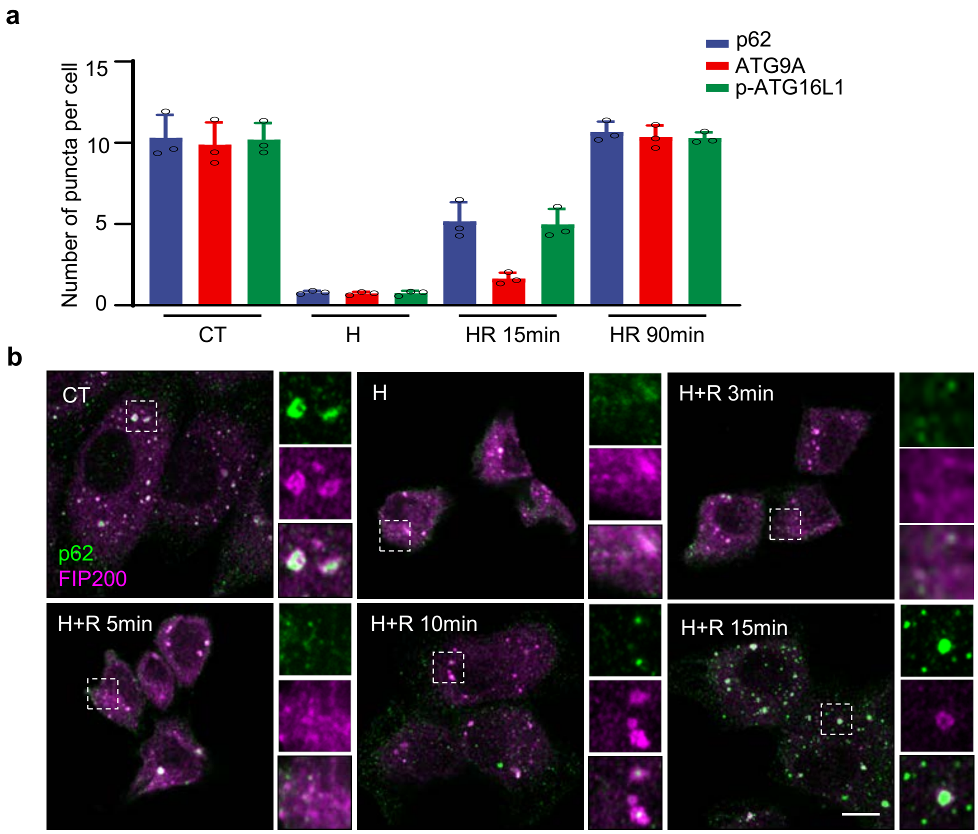

Supplementary Fig. 10 The recovery of p62 bodies after 1,6-hexanediol removal.

a, The number of indicated protein-positive puncta was quantified in Fig. 3i. Data are presented as mean ± SD, n=3 independent experiments; 100 puncta were assessed per independent experiment.

b, *Atg2ab* DKO cells were starved for 2 h and cells were treated with 2% 1,6-hexanediol for 20 min (H). The cells were then recovered by washing with PBS and incubation with complete medium (HR) for 3min, 5min, 10min or 15min. Then cells were stained with an antibody against p62 and FIP200. Scale bar, 10 μm.

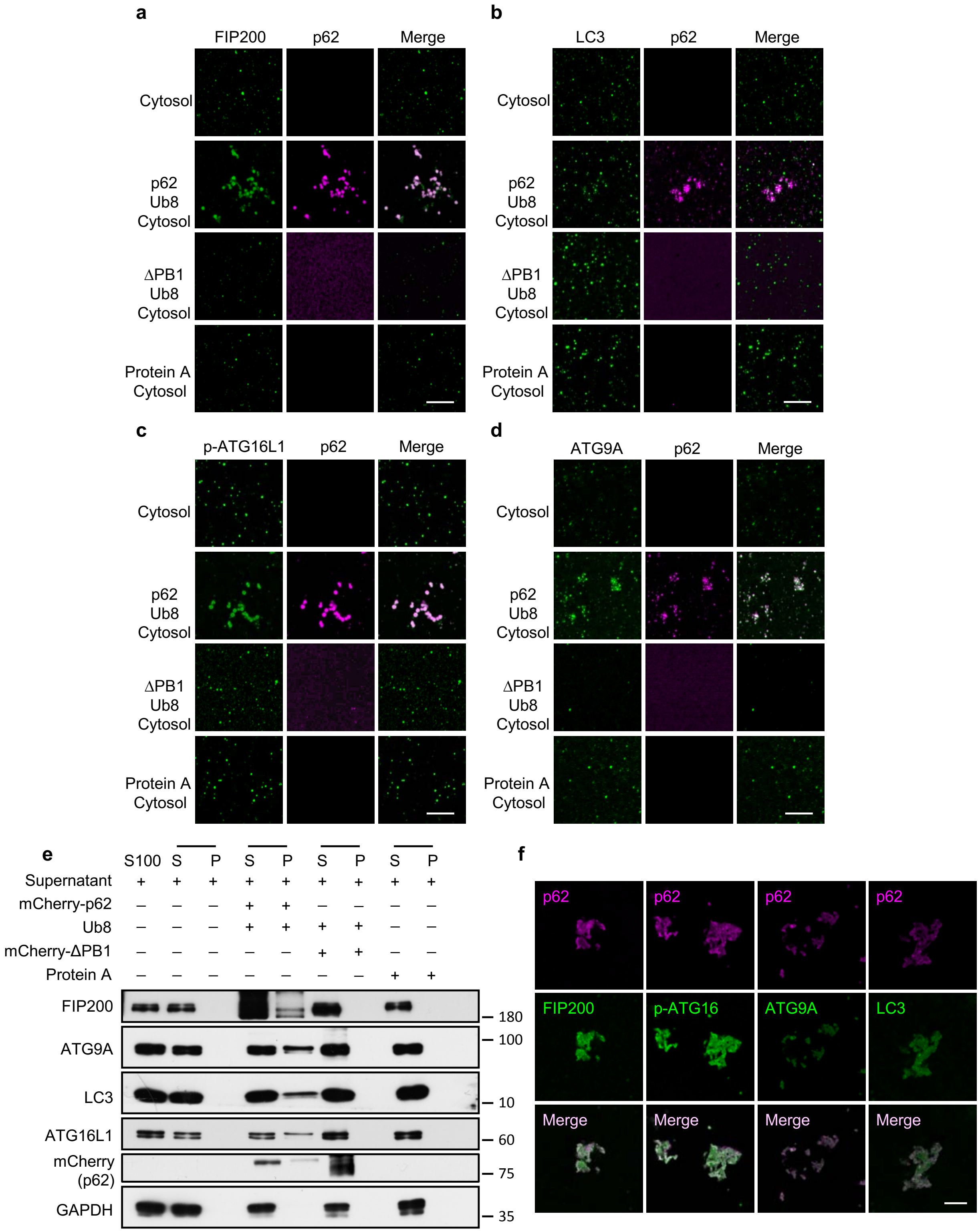

Supplementary Fig. 11 Recruitment of lipid membrane by p62 bodies *in vitro*.

a-d, p62 body and different protein /protein -interacting vesicles from Fig. 4c a were co-stained with antibodies against FIP200, ATG9A, ATG16L1 and LC3. Scale bar, 2 µm.

e, The sedimentation and supernatant from a were separated by centrifugation and analysed by western blot using antibodies against FIP200, ATG9A, ATG16L1, LC3, mCherry and GAPDH. S: supernatant, P: pellet.

f, p62 body and different protein-interacting vesicles from a were co-stained with antibodies against p62, FIP200, p-ATG16L1, ATG9A and LC3. Scale bar, 10 μm.

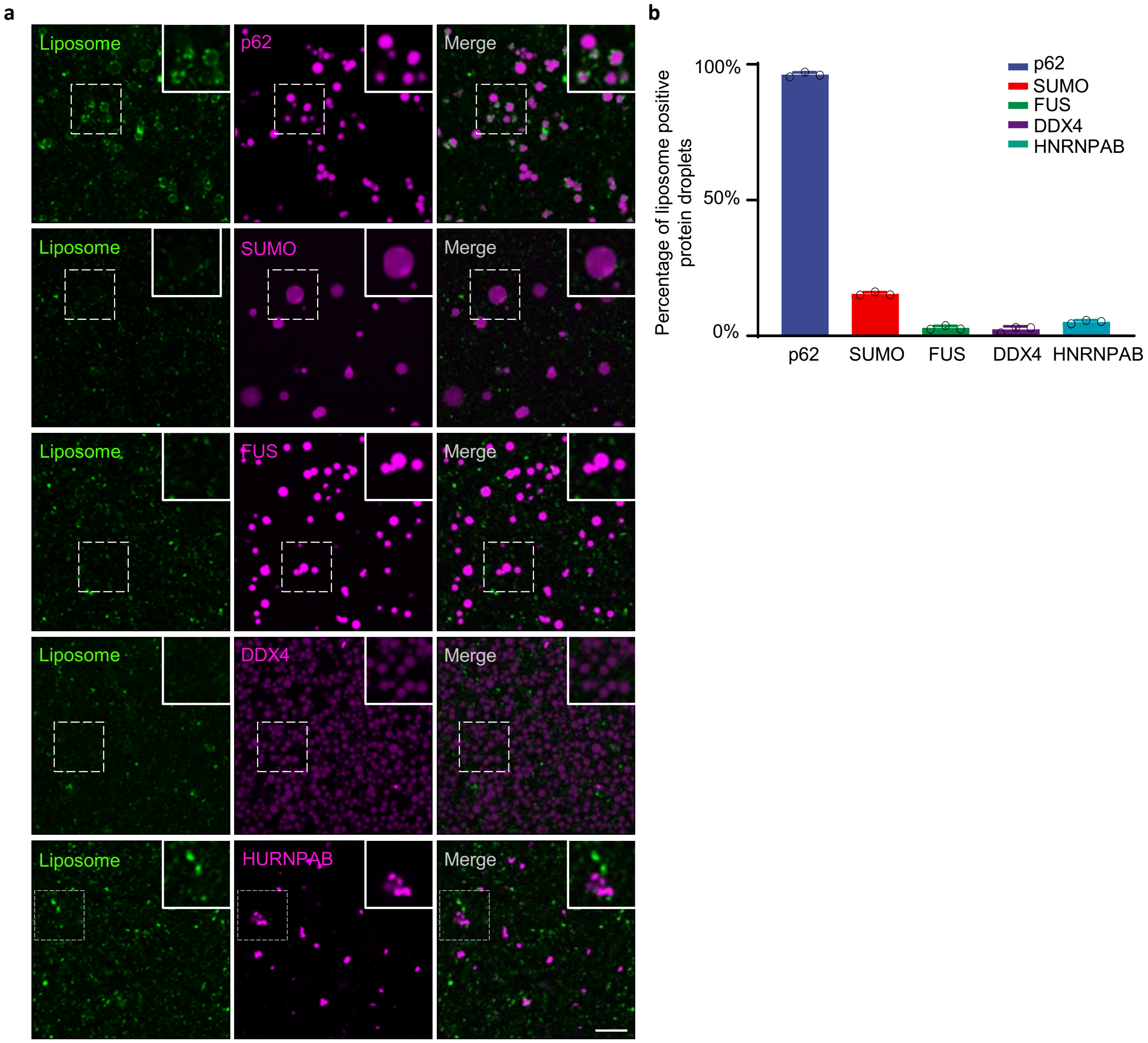

Supplementary Fig. 12 The recruitment of liposomes by different phase separated condensates.

a, Liposomes (PE-NDB) around the phase separated p62, SUMO, FUS, DDX4 and HURNPAB droplets were observed. Scale bar, 1 μm.

b, The percentage of p62 droplet colocalization with liposome was quantified in images from **a**. Data are presented as mean ± SD, n=3 independent experiments; 100 puncta were assessed per experiment.

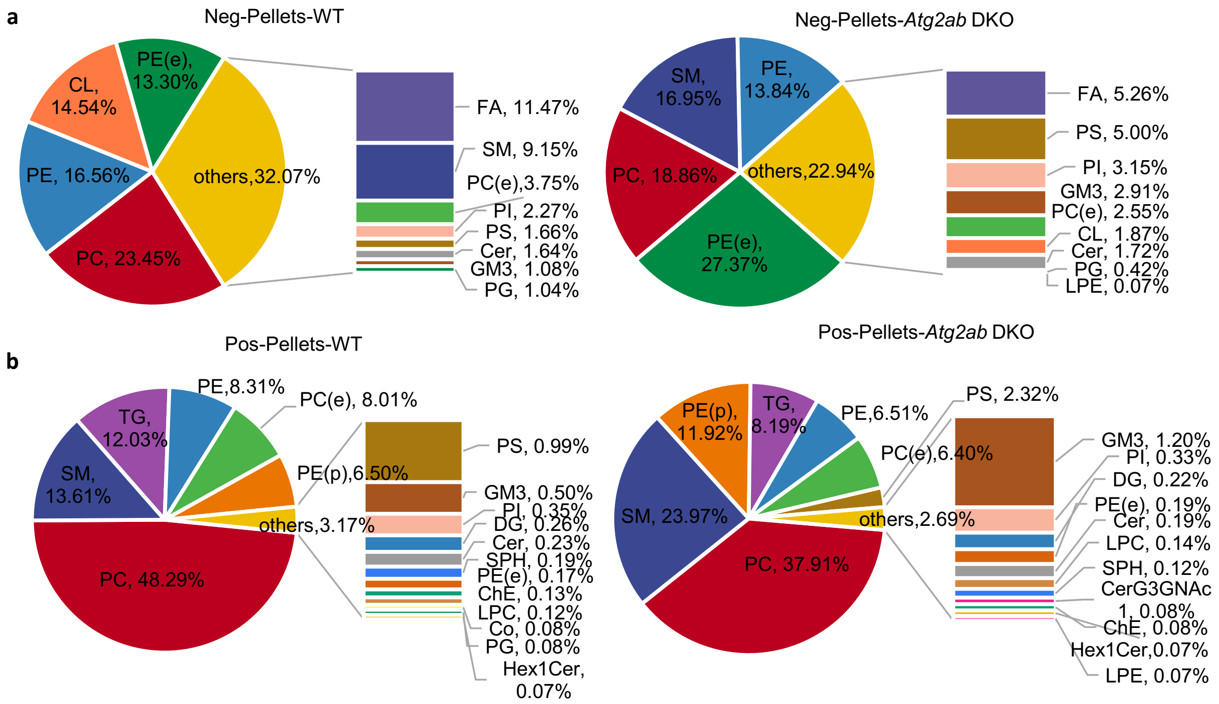

Supplementary Fig. 13 Lipidomic profiling of p62 bodies.

a-b, Composition of lipid classes that were associated with p62 bodies detected by liquid chromatography–mass spectrometry/mass spectrometry.

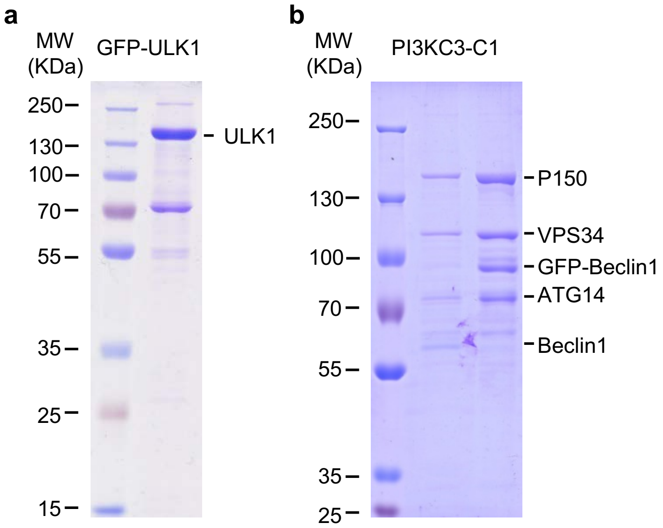

Supplementary Fig. 14 Purification of core autophagy proteins.
a, Coomassie brilliant blue stained SDS-PAGE showing the purified recombinant ULK1.
b, Coomassie brilliant blue stained SDS-PAGE showing the purified recombinant PI3KC3-C1.
