# Supplementary material for: Local membrane source gathering by p62 body drives autophagosome formation
Source: Nat Commun. 2023 Nov 13;14:7338. doi: 10.1038/s41467-023-42829-8 (PMC10643672; doi:10.1038/s41467-023-42829-8)
Supplement: Supplementary file 3 — Description of Additional Supplementary Files [file 41467_2023_42829_MOESM3_ESM.pdf]

Description of Additional Supplementary Files:

Supplementary Data 1: Proteome quantification of mixture (Mix), flow through (FT) and p62 droplet through LTQ Orbitrap Velos.

Supplementary Data 2: Significantly changed proteins of mixture (Mix), flow through (FT) and p62 droplet through ANOVA analysis.

Supplementary Data 3: Metascape enrichment network analysis of proteins enriched in the droplet *in vitro*.

Supplementary Data 4: Significantly changed proteins of APEX2-GFP-p62 through Student's T test.

Supplementary Data 5: Lipidomics profiling of p62 bodies by negative and positive modes MS analysis.
