# Supplementary material for: Local membrane source gathering by p62 body drives autophagosome formation
Source: Nat Commun. 2023 Nov 13;14:7338. doi: 10.1038/s41467-023-42829-8 (PMC10643672; doi:10.1038/s41467-023-42829-8)
Supplement: Supplementary file 9 — Reporting Summary [file 41467_2023_42829_MOESM9_ESM.pdf]

nature portfolio

Corresponding author(s): Na Mi

Last updated by author(s): Oct 12, 2023

Reporting Summary

Nature Portfolio wishes to improve the reproducibility of the work that we publish. This form provides structure for consistency and transparency in reporting. For further information on Nature Portfolio policies, see our Editorial Policies and the Editorial Policy Checklist.

Statistics

For all statistical analyses, confirm that the following items are present in the figure legend, table legend, main text, or Methods section.

n/a	Confirmed	
☐	☒	The exact sample size (*n*) for each experimental group/condition, given as a discrete number and unit of measurement
☐	☒	A statement on whether measurements were taken from distinct samples or whether the same sample was measured repeatedly
☐	☒	The statistical test(s) used AND whether they are one- or two-sided<br>*Only common tests should be described solely by name; describe more complex techniques in the Methods section.*
☒	☐	A description of all covariates tested
☒	☐	A description of any assumptions or corrections, such as tests of normality and adjustment for multiple comparisons
☐	☒	A full description of the statistical parameters including central tendency (e.g. means) or other basic estimates (e.g. regression coefficient) AND variation (e.g. standard deviation) or associated estimates of uncertainty (e.g. confidence intervals)
☐	☒	For null hypothesis testing, the test statistic (e.g. *F*, *t*, *r*) with confidence intervals, effect sizes, degrees of freedom and *P* value noted<br>*Give P values as exact values whenever suitable.*
☒	☐	For Bayesian analysis, information on the choice of priors and Markov chain Monte Carlo settings
☒	☐	For hierarchical and complex designs, identification of the appropriate level for tests and full reporting of outcomes
☐	☒	Estimates of effect sizes (e.g. Cohen's *d*, Pearson's *r*), indicating how they were calculated

Our web collection on statistics for biologists contains articles on many of the points above.

Software and code

Policy information about availability of computer code

Data collection	Nikon C2+ confocal microscope; Hitachi 7650B HT7800 transmission electron microscope; LABCONCO CentriVap; LTQ Orbitrap Velos.
Data analysis	Graph Pad prism 8.0;Fiji-win 64;NIS-Elements Viewer 5.21 64-bit;Microsoft Excel 2010; MaxQuant (1.5.38); Perseus (2.0.7).

For manuscripts utilizing custom algorithms or software that are central to the research but not yet described in published literature, software must be made available to editors and reviewers. We strongly encourage code deposition in a community repository (e.g. GitHub). See the Nature Portfolio guidelines for submitting code & software for further information.

Data

Policy information about availability of data

All manuscripts must include a data availability statement. This statement should provide the following information, where applicable:

- Accession codes, unique identifiers, or web links for publicly available datasets
- A description of any restrictions on data availability
- For clinical datasets or third party data, please ensure that the statement adheres to our policy

The mass spectrometry data generated in this study have been deposited in iProX (http://www.iprox.org/) under accession code IPX0005450000. Source data are provided with this paper.

Research involving human participants, their data, or biological material

Policy information about studies with human participants or human data. See also policy information about sex, gender (identity/presentation), and sexual orientation and race, ethnicity and racism.

Reporting on sex and gender	None of data and information were from human research participants.
Reporting on race, ethnicity, or other socially relevant groupings	Not applicable.
Population characteristics	Not applicable.
Recruitment	Not applicable.
Ethics oversight	Not applicable.

Note that full information on the approval of the study protocol must also be provided in the manuscript.

Field-specific reporting

Please select the one below that is the best fit for your research. If you are not sure, read the appropriate sections before making your selection.

[x] Life sciences   [ ] Behavioural & social sciences   [ ] Ecological, evolutionary & environmental sciences

For a reference copy of the document with all sections, see nature.com/documents/nr-reporting-summary-flat.pdf

Life sciences study design

All studies must disclose on these points even when the disclosure is negative.

Sample size	Sample size was not predetermined by statistic methods, but was selected according to the standard of relevant fields. For the quantification of immunofluorescence experiments, 50 to 100 cells per sample/condition were used for the analysis. For most cases, at least three independent experiments were performed.
Data exclusions	No data was excluded.
Replication	Unless specified, experiments were performed with three biological replicates, and all attempts at replication were successful.
Randomization	Randomization is not applicable in this study as no animal or clinical samples were involved.
Blinding	Investigators were not blinded as the readouts were not subjective.

Reporting for specific materials, systems and methods

We require information from authors about some types of materials, experimental systems and methods used in many studies. Here, indicate whether each material, system or method listed is relevant to your study. If you are not sure if a list item applies to your research, read the appropriate section before selecting a response.

Materials & experimental systems

n/a	Involved in the study	
[ ]	[x]	Antibodies
[ ]	[x]	Eukaryotic cell lines
[x]	[ ]	Palaeontology and archaeology
[x]	[ ]	Animals and other organisms
[x]	[ ]	Clinical data
[x]	[ ]	Dual use research of concern
[x]	[ ]	Plants

Methods

n/a	Involved in the study	
[x]	[ ]	ChIP-seq
[x]	[ ]	Flow cytometry
[x]	[ ]	MRI-based neuroimaging

Antibodies

Antibodies used	Primary antibodies:<br>mouse anti-GFP (Roche, # 11814460001, 1:500),<br>rabbit anti-LC3 (MBL, # PM036, # PM046, 1:500),<br>rabbit anti-ATG9A (abcam, # ab108338, 1:200),

mouse Anti-GM130 (BD,# 610822,1:300),
rabbit anti-actin (Sigma Aldrich, # A2066, 1:50000),
rabbit anti-p62 (MBL, # PM045, 1:1000),
mouse anti-p62 (MBL, # PM162-3, 1:1000),
rabbit anti-WIPI2 (abcam, # ab229225, 1:1000),
rabbit anti-ATG9A (abcam, # ab108338, 1:1000),
rabbit anti-ATG16L1 (abcam, # ab188642, 1:1000),
rabbit anti-p-ATG16L1 (abcam, # ab195242, 1:500),
rabbit anti-p-ATG14 (CST, # 92340S, 1:1000),
rabbit anti-ATG14 (MBL, # PD026MS, 1:1000),
rabbit anti-p-Beclin1 (CST, # 84966S, 1:1000),
rabbit anti-Beclin1 (abcam, # ab207612, 1:1000),
rabbit anti-ULK1 (abcam, # ab240916, 1:1000),
rabbit anti-mCherry (abcam,# ab 183628,1:1000),
mouse anti-ubiquitin (CST, # 3936S, 1:5000)
Secondary antibodies
Alexa Fluor 488 goat anti-rabbit IgG (Invitrogen, A11008,1:500),
Alexa Fluor 546 goat anti-rabbit IgG (Invitrogen, A11035,1:500),
Alexa Fluor 488 goat anti-mouse IgG (Invitrogen, A10680,1:500),
Alexa Fluor 546 goat anti-mouse IgG (Invitrogen, A11003; 1:500),
goat anti-rabbit (Southern Biotech, 4010-05; 1:5000),
goat anti-mouse (Southern Biotech, 1070-05;1:5000)

Validation

All the antibodies have been verified by the manufacturers according to the information on their websites

Eukaryotic cell lines

Policy information about

Cell line source(s)	The HEK239T and NRK cells were obtained from the American Type Culture Collection (ATCC).
Authentication	No authentication was performed.
Mycoplasma contamination	The cells were tested negative for mycoplasma contamination.
Commonly misidentified lines (See ICLAC register)	This study does not involve commonly misidentified cell lines.
